# Sandfly species diversity in association with human activities in the Kani tribe settlements of the Western Ghats, Thiruvananthapuram, Kerala, India

**DOI:** 10.1590/0074-02760140272

**Published:** 2015-04

**Authors:** Srinivasan Ranganathan, Subramanian Swaminathan

**Affiliations:** Vector Control Research Centre, Indian Council of Medical Research, Pondicherry, Pondicherry, India

**Keywords:** sandfly diversity, human activity, tribal settlements, the Western Ghats, Kerala, India

## Abstract

Sandfly prevalence in the Kani tribe settlements of Western Ghats in India was investigated. A total of 1,279 sandflies comprising 17 species was obtained. Sandfly abundance showed a negative correlation (r = -0.97, p = 0.003) with increase in altitudinal ranges from 0-1,000 m. When sandfly samples were grouped according to landscape characteristics of the location, the estimated Shannon-Weiner index (H) and species richness index (S) were high and species evenness index (J) was low in settlements located at 0-300 m altitudinal range. On the contrary, the values of H and J were high, while S was low at 301-600 m altitudinal range. With further increase in altitude, species diversity, S and J were low. Though the relative abundance of sandflies decreased with increase in altitude, the influence of altitudinal variation could not be attributed to determine sandfly diversity, since the number of sampling units were not uniform at all the altitudinal gradients due to nonavailability of suitable resting shelters. Sandfly species showed great aggregation at 0-300 m altitude interval, where not only the number of settlements were maximum (n = 19), but also the environmental conditions favoured sandfly abundance due to the concentration of tribal settlements, human dwellings and his activities.

Environmental conditions were conducive for the occurrence of diseases transmitted by haematophagous arthropods. Malaria, leishmaniases and viral encephalitis diseases propagated by blood sucking insects cause several millions of deaths among human in countries located in the tropical region (Jacobson et al. 2003). Leishmaniasis is an example of the diseases caused by flagellate parasites of the genus *Leishmania* and transmitted by female sandflies (Diptera: Psychodidae) (Killick-Kendrick 1990). These insects are adapted to a range of environments and conditions, with some species having wide geographical distribution, consequently occupying various biomes (Lewis 1978 , 1987, Dhanda et al. 1983, Killick-Kendrick 1983, Kalra & Bang 1988, Kumar et al. 1992, Srinivasan et al. 2013 ). The Indian subcontinent, which has diversified physiographic conditions, has many foci of visceral and cutaneous leishmaniasis due to sandfly abundance.

Distribution of sandflies in relation to ecological factors and altitude has been studied by several investigators in India. Ranjan et al. (2005) reported that soil, water table, temperature, annual rainfall, humidity and vegetation favour sandfly distribution. Bhunia et al. (2010) made an in-depth investigation and stated that in kala-azar endemic areas of Bihar in India the number of kala-azar cases decreased with increase in altitude (> 50 and 300 m) and beyond the altitude 300 m no kala-azar case was recorded, which implies that topography play a major role in determining sandfly distribution. Landform and land usage pattern have been reported to influence sandfly abundance in coastal areas of Pondicherry (Srinivasan et al. 2013). Recently, cutaneous leishmaniasis cases have been reported among the Kani tribe community, which is one of the Indian ethnicity or social groups settled in southernmost part of the Western Ghats, district of Thiruvananthapuram, state of Kerala (Simi et al. 2010, VCRC 2012). Srinivasan et al. (2014) described a new sandflies species from this region of the Western Ghats. As the area is known for cutaneous leishmaniasis transmission, a cross-sectional study was conducted to determine sandfly species composition and their abundance in relation to human activity in the tribal settlements.

## MATERIALS AND METHODS

The Western Ghats is a mountain range with reserve forests with an immense importance with unique biophysical and ecological feature. Scrub vegetation, deciduous vegetation, semi-ever green and ever green forests were found in the Western Ghats. At very high altitude grasslands occupied the hill top (Parthasarathy 1999, Sabu et al. 2008). Existence of the Western Ghats on the eastern side of Kerala in India has created a barrier across the path of the southwest monsoon. This had resulted in the creation of a significant climatic variation with abundant rainfall on the windward side and a dry belt on the lee eastern side. Temperature was relatively high during March-May and again in September. During that period temperature reached to a maximum of 33ºC which was considered less when compared with other states of India. The minimum temperature remained within 20ºC. Summer was followed by southwest monsoon that started pouring in June and continued till September. The northeast monsoon began in October and stopped at the end of December. Peak rain occurred in October. With the arrival of winter, there was a drop in the temperature. The winter lasted from November-January or February. There were great varieties of vegetation all along the Western Ghats: scrub jungles, dry and moist deciduous forests and semi-evergreen, evergreen forests and grassland (Parthasarathy 1999). Explorations conducted by the Kerala Forest Research Institute, Zoological Survey of India and other institutions brought out the wealth of the fauna and emphasised the need for further studies (Lewis 2012). The diversity of insect fauna of this region remained practically unknown, till the area came into the attention with the proposal of a hydel project (Gadgil 2012).

The Kani tribes were one of the Indian ethnicity or social groups who were settled in 28 settlements over southernmost part of the Western Ghats (08º37'49.7"N 077º11'29.7"E, 08º36'51.2"N 077º09'54.9"E, with altitude ranging from 0-1,000 m). The tribes were inhabiting in hut dwellings made of thatched roof (dry leaves, polythene sheets, tin sheets), walls with either dry plant materials, bamboo slots, sticks or polythene sheets, and a floor with mud or mud floor, each with one-two rooms (76.2%). The remaining tribes occupied in houses made of brick wall plastered with cement and rein-forced concrete cement roof (23.8%). Their traditional occupation included collection of nontimber forest products such as honey, bee-wax, medicinal plants, gums etc*. *All the tribal settlements were located in the difficult-to-reach area (VCRC 2012) and were under the control of Kuttichal Primary Health Care area, Nedumangadu, Thiruvananthapuram.

A cross sectional survey of sandflies was made in all the 28 tribal settlements, between November 2011-March 2012, which were conducive for sandfly survival due to optimum temperature and relative humidity (RH). Handheld mechanical aspirator collections were made indoors (human dwellings and cattle-sheds) and outdoors (tree holes, tree-buttresses, rock holes, rodent burrows) for collecting sandflies found on natural resting shelters. For this purpose, in each settlement, 10 samplings units, five number each, indoor and outdoor, were chosen based on random sampling method and sandflies were collected spending 10 min in each sampling unit, between 10:00 am 12:00 am. Light traps (each 5 number indoor and outdoor) were installed and activated between 05:00 pm-10:00 am using 12 watt batteries. Sticky traps (each 5 indoor and outdoor) made of white cardboard sheets (30 x 21 cm) and smeared with castor oil were placed indoors and outdoors at suspected breeding places of sandflies. Due to wild animal threat handheld aspirator collections were made after 10:00 am in the tribal settlements. Sandflies obtained in all collection methods were identified to species referring the standard keys (Artemiev 1978, Lewis 1978, Kalra & Bang 1988). The geographic coordinates were measured ~middle of each settlement, employing handheld Garmin GPS map 76 (Table I).

**TABLE I:** Study area: details of latitude, longitude, altitude and altitudinal ranges (m) of the Kani tribe settlements

Settlement number	Settlements	North latitude	East longitude	Altitude (m)	Altitudinal range
1	Kombidi	08°35’14.7”	077°07’40.1”	72	0-300
2	Podium	08°34’55.5”	077°07’27.3”	110	0-300
3	Kamalagam	08°34’55.5”	077°07’27.3”	110	0-300
4	Mela Amala	08°34’55.5”	077°07’27.3”	110	0-300
5	Patanipara	08°35’29.7”	077°09’30.6”	119	0-300
6	Chonambara	08°35’39.3”	077°09’09.9”	126	0-300
7	Kaithode	08°35’11.7”	077°07’50.7”	148	0-300
8	Mangode	08°35’11.7”	077°07’50.7”	148	0-300
9	Valpara	08°35’16.3”	077°10’15.2”	173	0-300
10	Ariyavila	08°35’08.5”	077°09’58.5”	174	0-300
11	Molamode	08°36’36.1”	077°10’18.7”	190	0-300
12	Erambiade	08°36’43.9”	077°11’22.5”	194	0-300
13	Monumukkam	08°35’56.8”	077°09’13.6”	197	0-300
14	Ploth	08°35’56.8”	077°09’13.6”	197	0-300
15	Panacavu	08°35’56.8”	077°09’13.6”	197	0-300
16	Keezheamala	08°35’18.5”	077°09’40.9”	204	0-300
17	Ayiramkal	08°33’48.3”	077°11’49.7”	217	0-300
18	Mukothivayal	08°33’48.3”	077°11’49.7”	217	0-300
19	Amode	08°33’10.8”	077°12’07.2”	223	0-300
20	Pothode	08°36’26.1”	077°11’36.3”	369	301-600
21	Cherumangal	08°36’26.1”	077°11’36.3”	369	301-600
22	Viavila	08°36’26.1”	077°11’36.3”	369	301-600
23	Anakkal	08°36’26.1”	077°11’36.3”	369	301-600
24	Pattampara	08°36’26.1”	077°11’36.3”	369	301-600
25	Kunnadi	08°36’26.1”	077°11’36.3”	369	301-600
26	Thannipara	08°34’56.6”	077°12’04.1”	439	301-600
27	Kunnatheri	08°35’50.2”	077°11’50.4”	739	601-800
28	Thottinpura	08°36’16.3”	077°11’58.4”	820	801-1,000
29	Baren land	08°36’18.3”	077°11’94.4”	1,001	1,000

The density of sandflies was calculated for each type of collection methods. Sandflies obtained using handheld aspirator were divided by the total number of man-hour (MHR) spent for collection and the density was expressed in number of sandflies per MHR. Similarly, the number of sandflies obtained from light trap and sticky trap collections were divided by the total number of traps used and the density was mentioned number of sandflies/light trap and number of sandflies/sticky trap, respectively.

Since the number of settlements was not uniform at each altitude to assess sandfly species diversity in relation to altitude, the elevation gradients of the Western Ghats of the tribal settlements were grouped into four categories: category I, 0-300 m with scrub vegetation, category II, 301-600 m with deciduous vegetation, category III, 601-800 m with semi-ever green, and category IV, 801-1,000 m with ever green forest. Data on soil type, canopy coverage and slope of the terrain published by Sabu et al. (2008) and Pascal et al. (2004) for the Western Ghats region were taken into consideration for analysis.

Sandfly species diversity in relation to altitude was evaluated. Shannon-Weiner index (H) and evenness index (J) were computed from the data obtained from different types of collections of sandflies in all the 28 tribal settlements, as follows: H = Σ Pi log_2 _Pi, where Pi is the proportion of total samples belonging to i-th species, J = H/ln (S), where S is the species richness, equal to the number of species in the total samples. The statistical analysis was performed in PAST (Hammer et al. 2001).

Even though sandfly diversity depends on S, to know whether sandflies in these tribal settlements were distributed evenly, a regression analysis was also made using H and S.

## RESULTS

A total of 1,279 sandflies was obtained from all the 28 settlements. Among the three collection methods, handheld aspirator collection yielded significantly (p < 0.05) more number of sandfly specimens (93.3%) followed by light trap (5.3%) and sticky trap catches (1.4%) (Table II). Seventeen species of sandflies (n = 1,279) were recorded*: Phlebotomus (Euphlebotomus) argentipes* (25.7%), *Phlebotomus (Anaphlebotomus) colabaensis *(2.9%), *Phlebo-* *tomus (Anaphlebotomus) stantoni *(1.6%), *Sergentomyia (Parrotomyia) baghdadis *(*50.7%*),* Sergentomyia (Parrotomyia) babu *(5.6%),* Sergentomyia (Neophlebotomus) zeylanica *(4.9%),* Sergentomyia (Neophlebotomus) arboris *(1.7%),* Sergentomyia (Parrotomyia) kauli *(1.6%), *Sergentomyia (Neophlebotomus) monticola *(1.2%),* Sergentomyia (Parrotomyia) barraudi *(0.9%),* Sergentomyia (Parrotomyia) jerighatiansis *(0.9%)* Sergentomyia (Neophlebotomus) malabarica *(0.6%),* Sergentomyia (Parrotomyia) rectangulata *(0.5%),* Sergentomyia (nic nic gr.) bailyi *(0.5%),* Sergentomyia (Parrotomyia) shorttii *(0.3%),* Sergentomyia (Neophlebotomus) dhandai *(0.2%) and* Sergentomyia (Sintonius) hospitii *(0.2%). *P. *(*Eup.) argentipes*, the proven vector of visceral leishmaniasis (kala-azar) in India, was found prevalent in 21 out of 28 settlements. All the 17 species were recorded indoors, among them, only 11 from outdoors.

**TABLE II:** Total number of sandflies obtained from different types of collections made in the Kani tribe settlements located at different altitudes

Type of collection	Habitat	Male (n)	Female (n)	Total n (%)
Light trap	Cattle shed	45	7	52 (4.1)
	Human dwelling	9	5	14 (1.1)
	Outdoor	0	1	1 (0.1)
Resting	Cattle shed	72	1	73 (5.7)
	Human dwelling	292	708	1,000 (78.2)
	Outdoor	51	69	120 (9.4)
Sticky trap	Indoor	5	3	8 (0.6)
	Human dwelling	0	0	0 (0)
	Outdoor	6	5	11 (0.9)

**Fig. 1:**
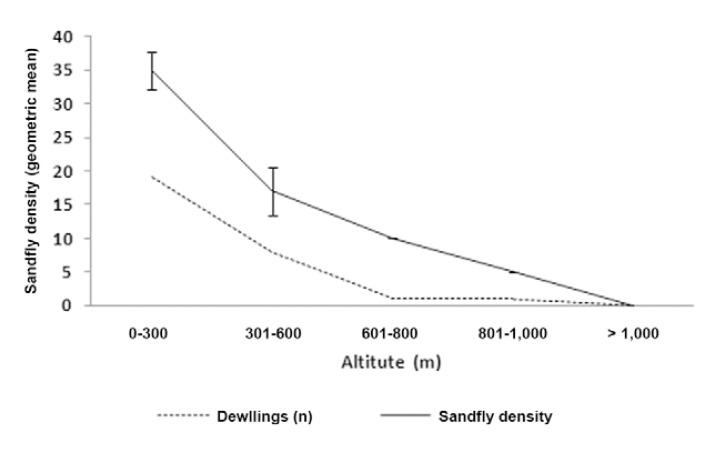
sandfly abundance in relation to altitude (m) and number of sandfly sampling units.

There were variations in the number of settlements at each altitudinal range. The number of settlements found at altitudinal ranges between 0-300 m, 301-600 m and 601-800 m, were 19, eight and one in number, respectively. At altitudinal ranges between 801-1,000 m and > 1,000 m, no settlement was found. A highest frequency of sandflies (n = 998) was captured in the settlements located at altitude ranging between 0-300 m, representing thrice the total captured specimens compared to those obtained in settlements located at altitudinal ranges between 301-600 m (n = 275), 601-800 m (n = 10) and 801-1,000 m (n = 5) (Fig. 1). No sandfly was obtained at > 1,000 m elevation. Of the 17 species recorded, 14 species were found up to an altitudinal range of 800 m, while *S. (Par.) baghdadis* and *S.* *(Par.) babu *were found up to 1,000 m altitude.

Density of sandflies obtained indoor and outdoor using handheld mechanical aspirator, light trap and sticky traps in settlements located at all altitudinal ranges were given in Table III. Although the number of sandflies collected was divided by the total MHR spent, mean density of sandflies obtained from settlements located at altitudinal ranges between 0-300 m was the highest. The increase in altitudinal ranges showed a decrease in sandfly density. Handheld aspirator collection yielded significantly high (p < 0.05) sandfly density than that of the light trap and sticky trap collections.

**TABLE III:** Density (mean ± standard deviation) of female sandflies obtained from different types of collections in Kani tribe settlements located at different altitudinal ranges

Altitudinal ranges	Hand-held aspirator (female number/man-hour)			Light traps (female number/light trap/night)			Sticky trap (female number/sticky trap)	
	Indoor	Outdoor		Indoor	Outdoor		Indoor	Outdoor
0-300 m	9.6 ± 8.6	1.2 ± 1.0		0.32 ± 0.2	0.72 ± 0.2		0.09 ± 0.1	0.32 ± 29
301-600 m	6.4 ± 9.0	0.9 ± 0.81		0.2 ± 0.91	0.2 ± 0.8		0.13 ± 0.1	0.15 ± 0.1
601-800 m	1.0	0.7		0.4	0.4		0	0.2
801-1,000 m	0.7	0.2		0	0		0	0.19
> 1,000 m	Nil	Nil		Nil	Nil		Nil	Nil

**Fig. 2:**
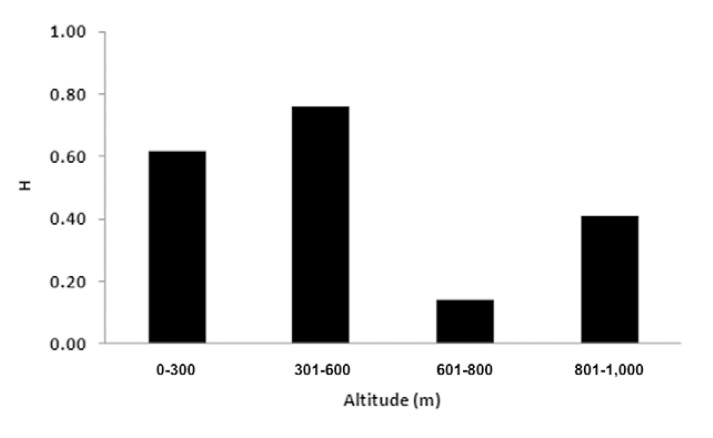
sandfly species Shannon-Weiner index (H) in relation to increase in altitude in southernmost part of the Western Ghats.

**Fig. 3:**
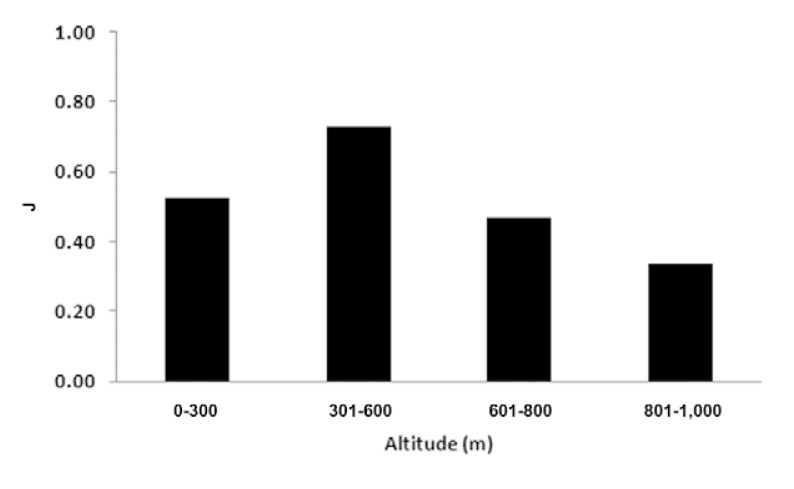
sandfly species evenness index (J) in relation to increase in altitude in southernmost part of the Western Ghats.

**Fig. 4:**
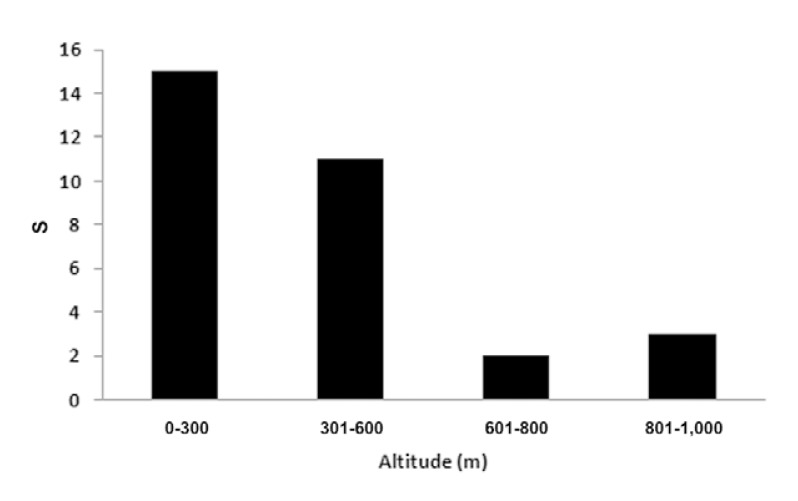
sandfly species richness index (S) in relation to increase in altitude in southernmost part of the Western Ghats.

The sandfly species diversity as indicated by the value of H, J and S among the settlements located at various altitudinal gradients (Figs 2-4) showed significant differences (r = -0.97, p = 0.003). In settlements located at the altitudinal range between 0-300 m, both species diversity and J were relatively low and S was high. On the other hand, species diversity and J were high and S was low at altitudinal range 301-600 m. With further increase in altitude (> 601 m) species diversity, J and S were very low.

**Fig. 5:**
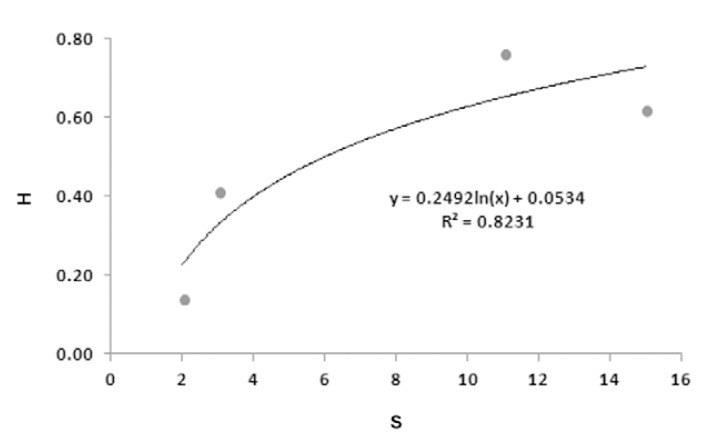
relationship between Shannon-Weiner index (H) and species richness index (S) in ecosystem disturbed by human activity is shown in logarithmic regression equation.

Although the relative abundance of sandflies showed a negative correlation (r = -0.97, p = 0.003) with increase in altitudinal ranges, altitudinal variations alone could not be attributed to determine sandfly diversity in the study, since the numbers of sandfly sampling units were not uniform at all the altitudinal gradients. The number of tribal settlements and number of human dwellings decreased with increase in altitude (r = -0.82, p = 0.001) due to lack of transport and communication, as the mountain region was difficult-to-reach. Human dwellings were found scattered in 19 settlements at the lower altitudinal gradient (0-300 m). S was high in the settlements located at lower altitudinal range. The reduction in H and J was due to irregular distribution of sandflies in the altitudinal range between 0-300 m (Fig. 5) where a particular sandfly species, i.e., *Se*. *(Par.)* *bagdhadis* dominated the sandfly community.

**Fig. 6:**
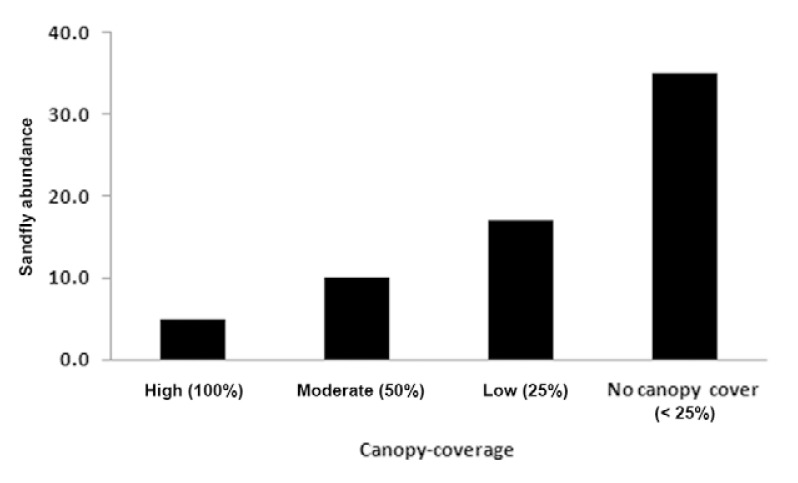
sandfly abundance in relation to canopy-coverage in southernmost part of the Western Ghats.

When the influence of soil type was analysed, sandflies were found to be abundant in settlements having humiferous soil [geometric mean (GM) = 35.0] followed by sandy soil (GM = 16) and red soil (GM = 5). Sandfly abundance showed a negative association with increase in mountain slope (Y = -9.6651x - 7.8912; R^2 ^= 0.9712). Forest canopy-coverage also influenced sandfly abundance to a greater extent (Fig. 6). Increase in canopy-coverage decreased sandfly abundance (Y = 9.7074x - 7.5174; R^2 ^= 0.9096) in the tribal settlements. Sandflies were found to be significantly abundant (*χ*² = 9891.5; p = 0.0001) (n = 998) in settlements located at altitude, ranging between 0-300 m, where the number of tribal settlements was also high (n = 19) compared to those located at higher altitudinal ranges (301-1,000 m). Therefore, the increase in the number of tribal settlements has influenced of environment due to human activities resulting in sandfly abundance.

## DISCUSSION

The Western Ghats is one of the biodiversity hot spots in the world. This mountain range mediates with the rainfall pattern in India by intercepting the southwestern monsoon winds and experiences heavy annual rainfall during the southwest monsoon from June-September and northeast monsoon from October-December. The wide variation in rainfall patterns coupled with the complex geography, particularly the altitude, produces a great variety of vegetation types (Parthasarathy 1999). Although forest vegetations, humiferous soil with leaf litter, ambient temperature, RH and heavy rain fall create ecological richness and provides conditions conducive for sandfly propagation and survival in this region, the number of sandflies caught in domestic and peridomestic habitats decreased with increase in altitudinal ranges. The capture of sandflies within the disturbed forest area located at 0-300 m altitudinal range was higher than the captures made at the areas located at 301-600 m and > 600 m altitudinal range.

As the mountain range is difficult-to-reach, the number of human settlements decreased with increase in altitude. Hence, most of the human settlements were located at lower altitudinal ranges. Therefore, human activities were confined to areas located at altitudes, ranging between 0-300 m. The environment was influenced with human activities for cultivation, which created new habitats, food sources and accumulation of organic materials, resulting in sandfly abundance. Besides, moisture content of the humiferous soil also increased due to irrigation, supporting sandfly breeding. Srinivasan et al. (2013) also authenticated the present finding. They have reported that fluvial land form supporting cultivation and luxuriant vegetation, favoured sandfly abundance and diversity in rural areas in southern India. However, the region located at higher elevations where the environment remained unaltered due to no or lesser human activity, sandfly-genic conditions were minimal, resulting in less sandfly abundance. Simsek et al. (2007) had also reported from southern Anatolia, Cukurova region of Turkey, that S of sandflies was highest (81%) at altitudinal range between 0-199 m compared to higher altitudinal ranges. Several such investigations carried out elsewhere also support the findings observed in the present study, where the distribution of sandflies showed negative correlation with increase in altitudinal range (Belen & Alten 2006, Simsek et al. 2007). Belen and Alten (2011) had also reported that sandfly abundance did not show any relationship with increase in altitude. Moreover, the geographical and ecological richness of the region provides numerous adult resting and larval sites for sandflies. It seems the altitude is one of several important factors that is necessary to predict the sandfly fauna in a region, but it is not a sufficient factor (Mohammad et al. 2013). Bhunia et al. (2010) reported that the number of kala-azar cases in endemic areas of Bihar decreased with increase in altitudinal range (50-300 m), which was indirectly evident that sandfly abundance diminished with increase in altitude. Further, they also correlated sandfly and sandfly-borne disease distribution with vegetation and found that low density vegetation or nonvegetative areas were found to be a favourable factor for sandfly abundance. Similarly, in the Kani tribe settlements sandfly abundance was high in no canopy-coverage areas.

Expansion of existing tribal settlements has resulted in the conversion of primary forest into secondary forest. Subsequently, Kani tribes altered the environment suitable for cultivation. This was possible, as the settlements receive rainfall throughout the year. Water clog made the soil and organic materials to retain moisture. Thus, a change in the terrain due to human activity changed ecological niches and conditions, favourable for sandfly proliferation. The agricultural activity due to scattering of food grains attracts rodents, which form ideal blood meal source for sandflies. Hence, sandfly abundance was found to be associated with human activities, as highest prevalence of sandflies was obtained in settlements located at the altitudinal ranges between 0-300 m. Cross et al. (1996) and Ghosh et al. (1999) had also observed abundant in areas which were in proximity to vertebrate host and cultivable areas. Further, Azevedo et al. (1996) and Sherlock (1996) had suggested that invasion of human into the forest enhances the risk of spread of sandfly-borne diseases, as zoophagic sandflies get adapted to anthropophagic feeding behaviour.

In the present investigation in tribal settlements located at lower altitudinal range, sandfly S was high and species diversity and J were low. Perhaps even distribution of ecological niche due to environmental change suiting to cultivation favoured a particular species to be more abundant, influencing species diversity. Hence, sandfly fauna was rich in this altitudinal range (0-300 m). Invasion of human in this part of the reserve forest region for domestication and cultivation supported sandfly-genic conditions, favouring sandfly abundance. In view of occurrence of cutaneous leishmaniasis cases among the Kani tribes (VCRC 2012), prevalence of sandflies with multiple hosts feeding pattern, including human (Sathish 2013), there is a risk of transmission of the cutaneous disease among the Kani tribes in the settlements. At this juncture, the investigation on sandfly diversity carried out in the Western Ghats region gains significance.
